# *Gadd45γ* and *Map3k4* Interactions Regulate Mouse Testis Determination via p38 MAPK-Mediated Control of *Sry* Expression

**DOI:** 10.1016/j.devcel.2012.09.016

**Published:** 2012-11-13

**Authors:** Nick Warr, Gwenn-Aël Carre, Pam Siggers, Jessica Vitos Faleato, Rachel Brixey, Madeleine Pope, Debora Bogani, Melissa Childers, Sara Wells, Cheryl L. Scudamore, Marianna Tedesco, Ivan del Barco Barrantes, Angel R. Nebreda, Paul A. Trainor, Andy Greenfield

**Affiliations:** 1 Mammalian Genetics Unit, Medical Research Council, Harwell, Oxfordshire OX11 0RD, UK; 2 The Mary Lyon Centre, Medical Research Council, Harwell, Oxfordshire OX11 0RD, UK; 3 Institute for Research in Biomedicine (IRB Barcelona) and Institució Catalana de Recerca i Estudis Avançats (ICREA), 08028 Barcelona, Spain; 4 Stowers Institute for Medical Research, 1000 East 50th Street, Kansas City, MO 64110, USA; 5 Royal Veterinary College, Hatfield, Hertfordshire AL9 7TA, UK; 6 Department of Anatomy and Cell Biology, University of Kansas Medical Centre, Kansas City, KS 66160, USA

## Abstract

Loss of the kinase MAP3K4 causes mouse embryonic gonadal sex reversal due to reduced expression of the testis-determining gene, *Sry*. However, because of widespread expression of MAP3K4, the cellular basis of this misregulation was unclear. Here, we show that mice lacking *Gadd45γ* also exhibit XY gonadal sex reversal caused by disruption to *Sry* expression. *Gadd45γ* is expressed in a dynamic fashion in somatic cells of the developing gonads from 10.5 days postcoitum (dpc) to 12.5 dpc. *Gadd45γ* and *Map3k4* genetically interact during sex determination, and transgenic overexpression of *Map3k4* rescues gonadal defects in *Gadd45γ-*deficient embryos. Sex reversal in both mutants is associated with reduced phosphorylation of p38 MAPK and GATA4. In addition, embryos lacking both p38α and p38β also exhibit XY gonadal sex reversal. Taken together, our data suggest a requirement for GADD45γ in promoting MAP3K4-mediated activation of p38 MAPK signaling in embryonic gonadal somatic cells for testis determination in the mouse.

## Graphical abstract

**Graphical Abstract:**

**Figure:**
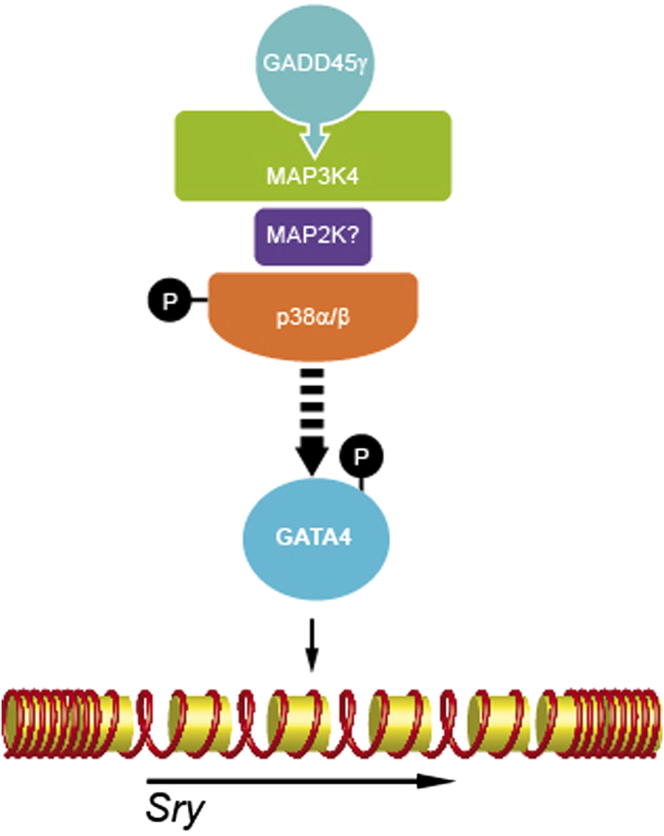

**Highlights:**

► Loss of *Gadd45γ* disrupts *Sry* expression and causes XY gonadal sex reversal ► *Gadd45γ* interacts with *Map3k4* during testis determination ► Loss of *Gadd45γ* and *Map3k4* disrupts p38 MAPK signaling and GATA4 phosphorylation ► Double-knockout embryos lacking *p38α* and *p38β* exhibit XY gonadal sex reversal

## Teaser

Testis determination in mammals is initiated by *Sry* expression. Warr et al. report XY gonadal sex reversal in mice lacking GADD45γ, a protein associated with cellular stress. Genetic and biochemical studies suggest a molecular pathway, linking GADD45γ, MAP3K4, p38 MAPK, and GATA4, required for the proper regulation of *Sry* expression.

## Introduction

MAP3K4 (also known as MEKK4) is a mitogen-activated protein kinase (MAPK) kinase kinase that acts in the stress-activated p38 MAPK and JNK signaling pathways to modulate a number of cellular pathways (Gerwins et al., 1997; Takekawa et al., 1997). We have previously shown that MAP3K4 is required for mouse testis determination on the C57BL/6J background and that chromosomally male (XY) embryos lacking functional MAP3K4 form gonads with an ovarian morphology at 14.5 days postcoitum (dpc) (Bogani et al., 2009). This gonadal sex reversal is associated with reduced expression of *Sry* in the supporting cell lineage during a critical window for testis determination. This disruption to *Sry* expression in *Map3k4*^−/−^ mutants results in failure to execute the testis-determining pathway of gene expression and, thus, absence of Sertoli cell differentiation and testis cord formation. These observations established *Map3k4* as an autosomal testis-determining gene, but the cellular basis of the misregulation of *Sry* expression remained unclear in this earlier study. Disruption to cell proliferation in supporting cell precursors might arguably cause a reduction in the number of SRY-positive cells observed in mutant gonads, causing reduced *Sry* expression. However, cell-autonomous defects in the transcriptional regulation of *Sry* in pre-Sertoli cells might also account for reduced cell numbers, given the proposed role of SRY in recruitment of additional cells to the Sertoli cell fate (Sekido and Lovell-Badge, 2009). Widespread expression of MAP3K4 means that disruptions to either of these processes might be the primary cause of the gonadal sex reversal.

To better understand how MAPK signaling regulates sex determination in the mouse in vivo, we sought molecules regulating this process that had a higher degree of cellular and temporal specificity of expression. While MAP3K4 is essentially ubiquitous, its activation may be regulated in a precise spatiotemporal fashion (Winter-Vann and Johnson, 2007). MAP3K4 interacts promiscuously with different classes of protein, including GADD45 (growth arrest and DNA damage response) protein family members (Takekawa and Saito, 1998). Such protein partners of MAP3K4 are candidate regulators of its embryonic gonadal activity.

*GADD45* was identified as a gene upregulated by agents that cause DNA damage (Fornace et al., 1988). Three related proteins, GADD45α, GADD45β, and GADD45γ, were subsequently identified in yeast two-hybrid screens for proteins that interact with MAP3K4 (Takekawa and Saito, 1998). This family of small, acidic nuclear proteins has been implicated in a range of biological processes, including cell cycle progression and differentiation, DNA repair, and active DNA demethylation (Barreto et al., 2007; Hollander and Fornace, 2002; Niehrs and Schäfer, 2012). In the context of MAPK signaling, GADD45γ activates the p38 MAPK and JNK pathways in T cells (Lu et al., 2001) and GADD45 proteins have been shown to activate MAP3K4 by disrupting an autoinhibitory domain of MAP3K4, inducing autophosphorylation by formation of an active dimer (Miyake et al., 2007). Significantly, GADD45β and GADD45γ have been shown to act in a common pathway with MAP3K4, regulating IFNγ production in T cells in vitro (Chi et al., 2004). Despite these data, however, the precise physiological functions of GADD45 proteins are not well characterized: GADD45γ, for example, has been reported to be dispensable for normal mouse development on a mixed genetic background (Hoffmeyer et al., 2001).

Here we report that *Gadd45γ* is required for testis determination in mice and describe data supporting a model in which GADD45γ functions in the same pathway as MAP3K4 and p38 MAPK during gonadogenesis. *Gadd45γ* transcription is initiated at very early stages of gonad development in somatic cells of both XY and XX embryos, its spatiotemporal expression profile bearing a striking resemblance to that of *Sry*. Adult C57BL/6J XY mice lacking *Gadd45γ* exhibit fully penetrant gonadal sex reversal caused by reduced embryonic expression of *Sry* at the transcript and protein level from around 10.75 to 11.0 dpc. We show that, despite its known role in active DNA demethylation, the methylation profile of CpG dinucleotides just 5′ to the *Sry* transcription start site in gonadal somatic cells is unaffected in mutants. However, we show that disruption to *Sry* expression in *Gadd45γ* and *Map3k4* null mutants is associated with reduced levels of activated (phosphorylated) p38 MAPK in embryonic gonads at around 11.25 dpc. Moreover, phosphorylation of the known testis-determining protein, GATA4, is also reduced. Finally, we demonstrate that simultaneous inactivation of the genes encoding the p38α and p38β MAPKs also causes embryonic XY gonadal sex reversal due to reduced levels of *Sry* expression. We propose that GADD45γ positively regulates MAP3K4-mediated p38 MAPK signaling from around 10.5 dpc in XY embryonic gonadal somatic cells as a requirement for testis determination.

## Results

### *Gadd45γ* Is Expressed in the Newly Formed Gonad with a Spatiotemporal Profile Reminiscent of *Sry*

**Figure 1:**
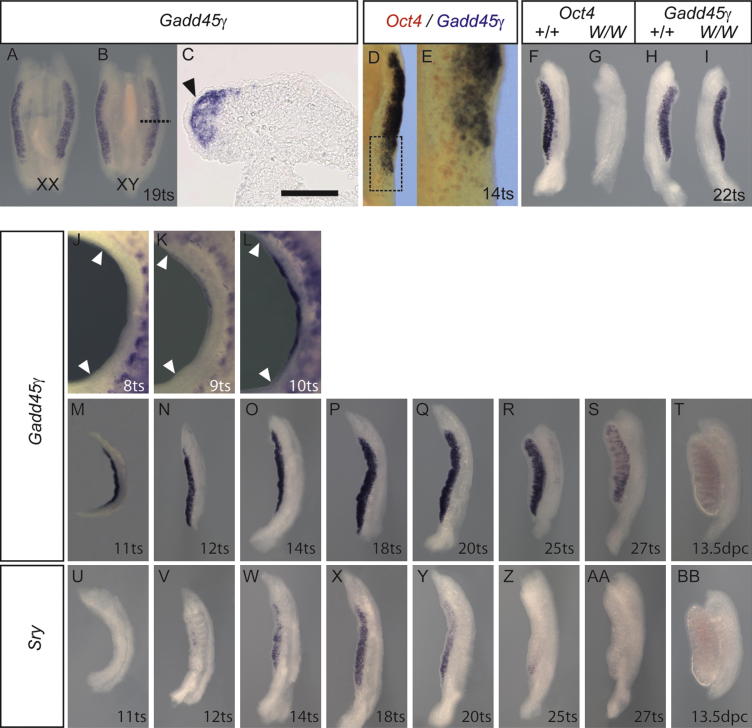
*Gadd45γ* Is Expressed in Somatic Cells of the Developing XX and XY Gonad with a Spatiotemporal Profile Reminiscent of *Sry* (A–C) WMISH in the developing XX (A) and XY (B) wild-type gonads at 11.5 dpc (19 ts). Expression is restricted to the body of the gonad and not detected in the coelomic epithelium (C, arrowhead). Dotted line in (B) indicates the position of the section shown in (C). Scale bar in (C) is 100 μm. (D and E) Two-color WMISH with *Oct4* (red) and *Gadd45γ* (blue). (F–I) *Oct4* is not detected in *W/W* homozygous gonads at 11.5 dpc (F and G), in contrast to *Gadd45γ* expression (H and I). (J–L) *Gadd45γ* is first detected at 9 ts, in the central portion of the gonad (J and K); white arrowheads mark anterior and posterior limits of the gonad in (J–L). By 10 ts expression is detected throughout the gonad (L). (M–T) *Gadd45γ* is expressed at high levels until around 25 ts (M–R), and declines thereafter, being undetectable at 13.5 dpc (S and T). (U–BB) *Sry* expression profile in the same genetic background.

To identify potential regulators of MAP3K4 in the embryonic gonad and shed light on its cellular function, we studied expression of several genes implicated in the MAPK signaling pathway. We examined the expression of *Gadd45γ*, which encodes a known MAP3K4-interacting protein, using whole mount in situ hybridization (WMISH) (Figure 1). At 11.5 dpc, we observed prominent expression of *Gadd45γ* in the developing gonad of both XY and XX gonads (Figures 1A and 1B). Sectioning of tissues revealed that this expression was restricted to cells in the body of the gonad and absent from the coelomic epithelium (Figure 1C), a source of Sertoli cell progenitors (Karl and Capel, 1998). Comparison between the expression of *Gadd45γ* and *Oct4,* a primordial germ cell marker, at 11.25 dpc indicated that each is expressed in a distinct subset of gonadal cells, suggesting that *Gadd45γ* is not germ cell dependent (Figures 1D and 1E). To confirm this, we used gonads from embryos homozygous for the *W* allele of *c-kit*, which lack germ cells (Russell, 1979) (Figures 1F–1I). There was no difference in *Gadd45γ* signal detected between *W/W* mutant and wild-type gonads after WMISH (Figures 1H and 1I), in contrast to the complete loss of the germ cell marker *Oct4* in mutants (Figures 1F and 1G). Therefore, we conclude that *Gadd45γ* expression is primarily restricted to somatic cells of the gonad, which at this stage includes supporting cell precursors and other progenitor lineages. Careful profiling of *Gadd45γ* revealed an onset of detectable expression at around nine tail somites (ts) (approximately 10.5 dpc) in cells toward the center of the gonad (Figures 1J and 1K). This expression then spreads and is visible in all regions of both XY and XX gonads by 10–11 ts (Figures 1L and 1M). *Gadd45γ* expression is maintained subsequently in XY gonads until the appearance of testis cords (around 25 ts), after which transcript levels drop (Figures 1N–1S). Expression is negligible by 13.5 dpc (Figure 1T). This spatiotemporal expression profile, including the center-to-pole spatial expansion and the narrow temporal window, is strikingly similar to that of *Sry* (Figures 1U–1BB). However, we consistently detected *Gadd45γ* at 9–10 ts, earlier than *Sry*, which is detectable from around 12 ts using WMISH (Figure 1V). The pattern of *Gadd45γ* expression in testis cords of 12.5 dpc gonads was reminiscent of a Sertoli cell marker such as *Sox9*, but expression levels were too low at this stage to detect clear signal in sectioned material. We were also unable to detect endogenous GADD45γ protein with a range of antibodies tested (data not shown). However, the spatiotemporal profile of *Gadd45γ* described here is consistent with its acting in the same somatic cell lineage as *Sry* and, given its earlier detectable expression, potentially contributing to regulation of *Sry* expression.

### Loss of *Gadd45γ* Causes Gonadal Sex Reversal in Adult XY Mice

**Figure 2:**
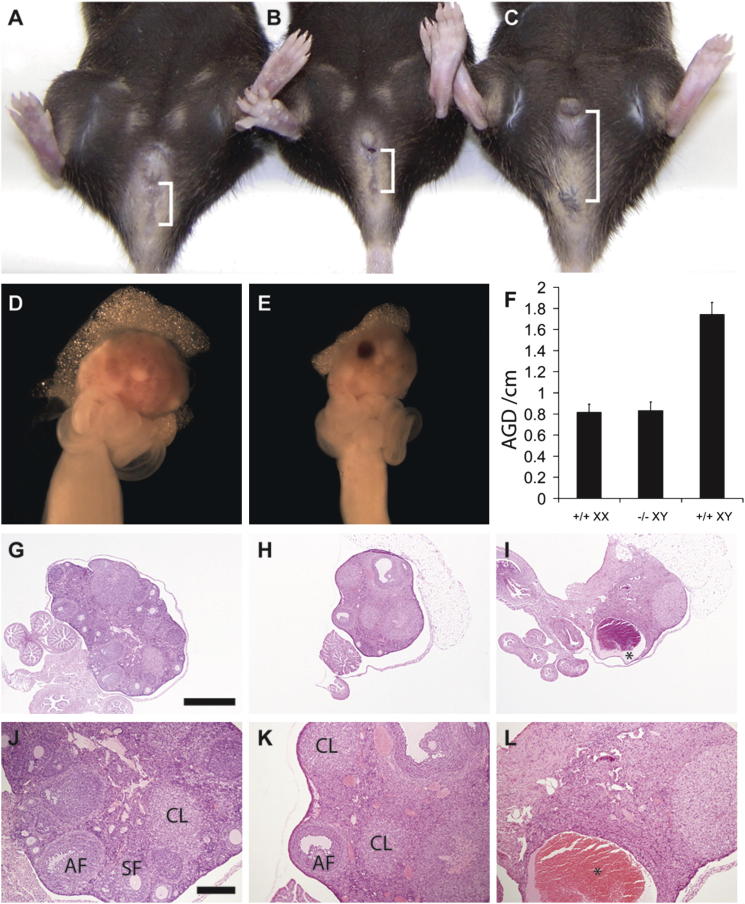
XY Mice Lacking *Gadd45γ* Are Phenotypic Females (A–C) External genitalia of wild-type XX (A), *Gadd45γ*^−*/*−^ XY (B), and wild-type XY (C) adult mice. (D–F) Mutant XY ovaries (E) appear smaller than their wild-type XX counterparts (D), and some variation in morphology is apparent. Anogenital distance (square brackets) is quantified (F). (G–L) Histological examination reveals that some mutant XY ovaries (H and K), have an almost normal appearance when compared to XX controls (G and J), while others are very hypoplastic with very few or no follicles (I and L). Cysts (asterisks, I and L) were commonly detected in XY mutant ovaries. Scale bar in (G), 1 mm; scale bar in (J), 300 μm. AF, antral follicle; SF, secondary follicle; CL, corpus luteum. Error bars, SEM.

To test whether GADD45γ functions, like MAP3K4, to regulate testis determination, we examined sexual development in *Gadd45γ*-deficient mice bred on the C57BL/6J (B6) genetic background, which is sensitized to disruptions to testis development (Bogani et al., 2009; Bouma et al., 2007; Warr and Greenfield, 2012). We first compared chromosomal and phenotypic sex in a cohort of homozygous mutant animals at 8 weeks of age. Remarkably, all XY homozygotes had been scored as phenotypic females at weaning. Anogenital distance in these mice was comparable to wild-type female controls (Figures 2A–2C and 2F). In matings with wild-type males, sex-reversed mice gave copulatory plugs indicative of normal mating behavior but were infertile, yielding no offspring. Microscopically, reproductive tracts from XY *Gadd45γ*^−*/*−^ mice had a predominantly female phenotype consisting of identifiable ovaries and oviduct and uterine structures. Although the ovaries were universally smaller than those of wild-type controls (Figures 2D and 2E), the ovarian morphology was quite variable between individuals (Figures 2G–2L). In some animals, there were very few primordial, primary, and secondary follicular structures with no follicles developing to the antral stage and occasional cystic structures (Figures 2I and 2L). In other animals, a full complement of follicular stages and corpora lutea from multiple cycles was present (Figures 2H and 2K), in association with an active uterine endometrium, suggesting coordinated reproductive hormonal stimulation. No overt abnormalities were observed in XY heterozygous animals. XX *Gadd45γ*^−*/*−^ mice were fertile females that were used to generate additional mutant mice.

### The Expression of *Sry* and Downstream Testis-Determining Genes Is Disrupted in XY *Gadd45γ*^−*/*−^ Embryonic Gonads

**Figure 3:**
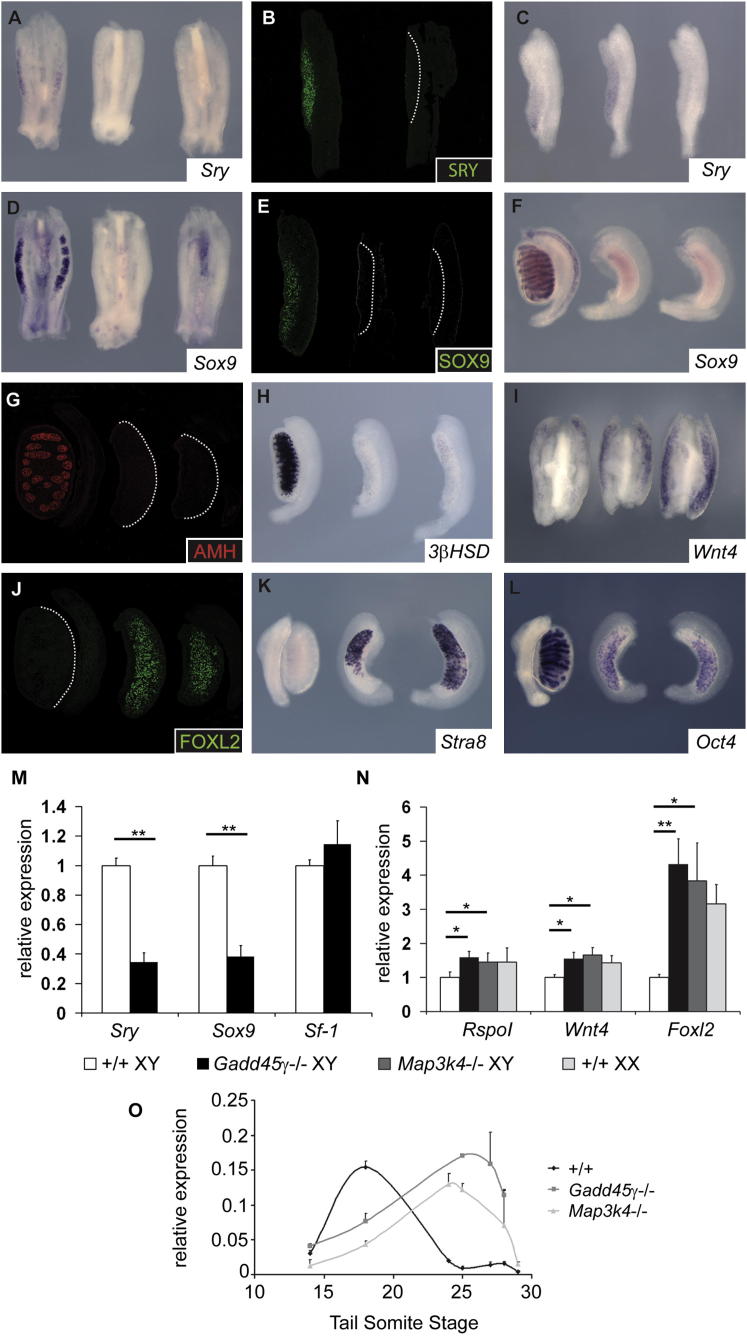
XY Gonadal Sex Reversal in *Gadd45γ*-Deficient Embryos Is Caused by Disrupted *Sry* Expression (A–L) Frames containing three gonadal tissue samples have the order, from left to right, XY wild-type, XY *Gadd45γ*^−*/*−^, and XX wild-type. WMISH reveals reduction in *Sry* transcripts detected at 11.25 dpc (16 ts) in XY mutants (A). SRY protein levels are similarly diminished in mutants (B, mutant on right). By 12.5 dpc, *Sry* expression is detectable at higher levels in the mutant XY gonad, in contrast to negligible levels in the XY wild-type (toward posterior of gonad) and XX control at the same stage (C). *Sox9* transcripts are undetectable in mutant gonads at 18 ts (D) and 14.5 dpc (F), and SOX9 protein is also not detected at 11.5 dpc (E). AMH is also undetectable in mutant XY gonads at 14.5 dpc (G), and *3βHSD* is absent at 13.5 dpc (H). *Wnt4* is expressed in mutant XY gonads at 11.5 dpc (I). FOXL2 and *Stra8* are expressed at high levels in mutant XY gonads at 14.5 dpc (J and K, respectively), concomitant with downregulation of *Oct4* expression (L). (M–O) Loss of *Sry* and *Sox9* expression in XY mutant gonads was confirmed by qRT-PCR (M) at 18 ts, as was inappropriate expression of ovarian marker genes (N). qRT-PCR reveals a delay in *Sry* expression in *Gadd45γ*^−*/*−^ and *Map3k4*^−*/*−^ gonads (O). Error bars, SEM. Dotted white lines mark the boundary between gonad and mesonephros in samples that do not exhibit significant expression of marker protein. See also Figure S1 and Figure S2 for additional phenotypic analyses of *Gadd45γ*^−*/*−^ and *Map3k4*^−*/*−^ embryonic gonads.

To address the cause of sex reversal in XY *Gadd45γ*^−/−^adult mice, we examined gene expression at the sex-determining stage of gonad development, beginning with *Sry*. WMISH at 16 ts revealed greatly reduced *Sry* signal in *Gadd45γ*^−/−^ mutant gonads when compared to wild-type controls (Figure 3A). This reduction in levels of *Sry* transcript was validated by quantitative RT-PCR (qRT-PCR) analysis of urogenital ridges at 11.5 dpc (Figure 3M). Quantitation indicated an approximate 3.5-fold reduction in *Sry* levels in mutant gonads at this stage. Expression of *Sry* was subsequently detected in *Gadd45γ*^−/−^mutant gonads at 26 ts (around 12.5 dpc), indicating a significant delay in expression (Figures 3C and 3O). We also examined SRY protein localization by immunostaining, and this identified large numbers of SRY-positive pre-Sertoli cells in wild-type gonads at 11.5 dpc (18 ts) but very few positive cells in mutant XY gonads at the same stage when imaged with identical settings (Figure 3B). When combined with the observed expression profile of *Gadd45γ*, which is expressed in somatic cells in the body of the gonad and not in the coelomic epithelium, these data suggest that GADD45γ is required for appropriate regulation of *Sry* expression in pre-Sertoli cells. We also observed that cellular proliferation was normal in XY *Gadd45γ* mutant gonads at 11.0 dpc (Figure S1 available online), a time at which cell proliferation is key to testis determination (Schmahl and Capel, 2003). This suggests that disruption to *Sry* expression occurs as a result of cell-autonomous functions of GADD45γ, rather than originating in, for example, disruption to the proliferative potential of a putative supporting cell precursor lineage in the coelomic epithelium (Karl and Capel, 1998).

Expression of a second key sex-determining gene, *Sox9*, was virtually undetectable in mutant XY gonads at 11.5 dpc and 14.5 dpc (Figures 3D–3F). Another Sertoli cell marker, AMH, was also absent at 14.5 dpc (Figure 3G), as was the Leydig cell marker, *3β-HSD* (Figure 3H). In contrast, the ovarian pathway of gene expression was activated, with prominent expression of *Wnt4* at 11.5 dpc (Figure 3I). The ovarian somatic marker, FOXL2, was also strongly induced in supporting cells of the XY mutant gonads at 14.5 dpc (Figure 3J). This activation of the ovarian pathway is predicted based on the mutual antagonism that exists between the testis- and ovary-determining gene regulatory networks (Kim et al., 2006; Warr and Greenfield, 2012). At 14.5 dpc, *Stra8* was strongly expressed in sex-reversed XY gonads, in contrast to XY controls (Figure 3K), indicating the presence of large numbers of meiotic germ cells. This is also supported by the diminution of *Oct4* expression, a marker of pluripotency, at 14.5 dpc in XY *Gadd45γ* ^−/−^gonads and XX controls, in contrast to XY wild-type gonads (Figure 3L). qRT-PCR analyses at 18 ts validated the inappropriate activation of ovary-determining genes in mutant XY gonads observed by WMISH (Figure 3N). Overall, these data suggest an early failure to execute the testis-determining program due to greatly reduced *Sry* expression during a critical time window (11–11.25 dpc) and a consequent activation of the ovary-determining pathway (Hiramatsu et al., 2009; Kim et al., 2006).

### Genetic Interactions between *Gadd45γ* and *Map3k4* during Testis Determination

**Figure 4:**
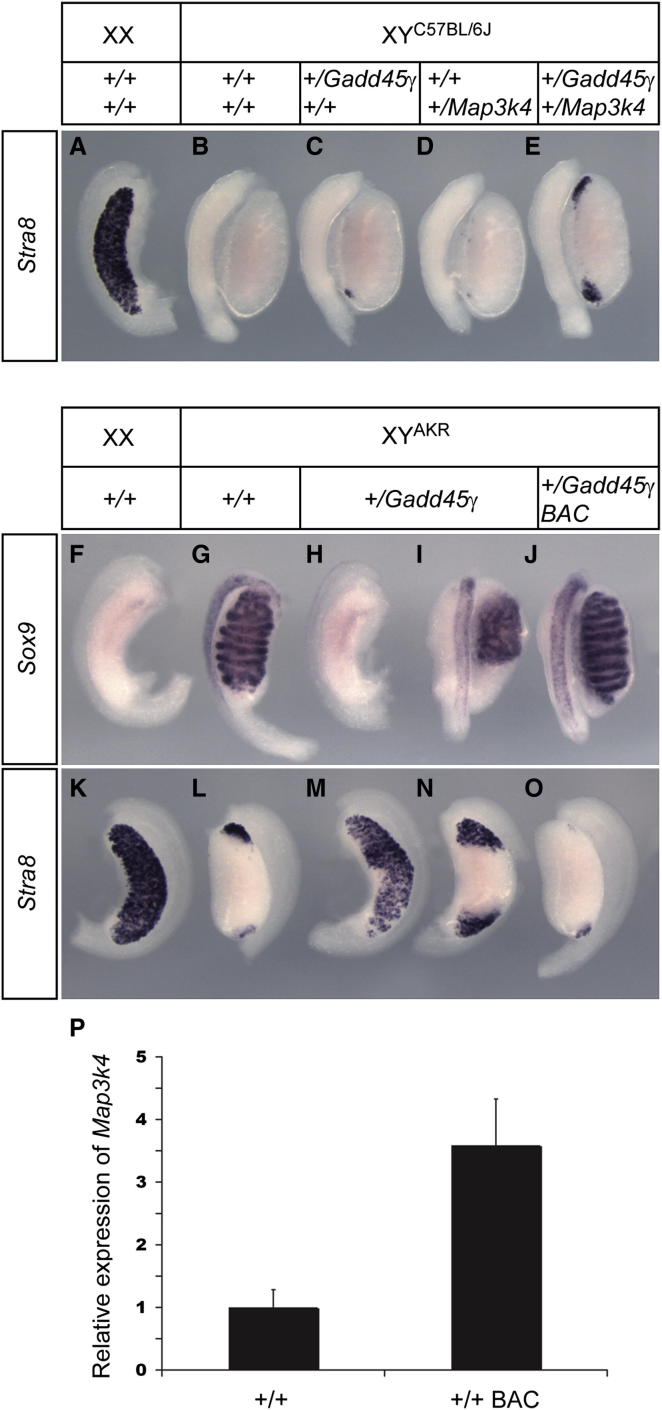
Genetic Analyses of Functional Interactions between *Gadd45γ* and *Map3k4* during Testis Determination (A and B) *Stra8* expression is ovary specific in wild-type gonads at 14.5 dpc. (C–O) Significant numbers of *Stra8*-positive cells are detectable at poles of XY gonads lacking one copy of both *Gadd45γ* and *Map3k4* (E). Only very few *Stra8*-positive cells are detected, occasionally, in singly heterozygous gonads (C and D). *Sox9* and *Stra8* WMISH reveal ovary development (H and M), or ovotestis development (I and N), in embryos lacking a single copy of *Gadd45γ* on C57BL/6J-Y^AKR^ (B6-Y^AKR^), in contrast to wild-type controls (G and L). Wild-type XX littermate controls do not express *Sox9* (F) but express high levels of *Stra8* (K). The presence of a *Map3k4* BAC transgene rescues testis development in *Gadd45γ*^*+/−*^ heterozygotes (J and O). (P) Quantitation of gonadal *Map3k4* expression by qRT-PCR in wild-type (+/+) and BAC transgenic (+/+ BAC) embryos at 11.5 dpc indicates an approximate 3.5-fold increase of expression in BAC transgenics. Error bars, SEM.

The identical gonadal phenotypes of mice lacking GADD45γ and MAP3K4 suggest that a *Gadd45γ*-*Map3k4* pathway may function in vivo to regulate testis determination. To investigate this further, we performed loss-of-function and gain-of-function genetic experiments. First, we performed a conventional genetic interaction test by generating embryos lacking a single copy of both *Gadd45γ* and *Map3k4*. Gonads dissected from doubly heterozygous XY embryos at 14.5 dpc were assessed for morphology and scored as testes (Figures 4A–4E). However, closer examination of these using the marker *Stra8* revealed significant numbers of meiotic germ cells at the gonadal poles (Figure 4E), suggesting a delay in the receipt of the testis-determining signal to these regions and subsequent local activation of the ovarian pathway. In contrast, in single heterozygous embryos, we observed very few *Stra8*-positive cells at the poles and in only a small fraction of embryos (Figures 4C and 4D). In wild-type XY gonads, *Stra8*-positive cells were not detected (Figure 4B). Thus, a subtle but clear genetic interaction occurs between *Gadd45γ* and *Map3k4* during testis determination.

We examined the dosage sensitivity of *Gadd45γ* further by analyzing embryos lacking a single copy of the gene on the C57BL/6J-Y^AKR^ (B6-Y^AKR^) genetic background. B6-Y^AKR^ is highly sensitized to further disruption to testis determination (Bouma et al., 2007), including loss of a single copy of *Map3k4* (Bogani et al., 2009). We found that B6-Y^AKR^ embryos lacking a single copy of *Gadd45γ* showed severe disruption to testis determination (Figures 4F–4I and 4K–4N). Around 50% of heterozygous mutant embryos formed gonads with an ovarian morphology at 14.5 dpc, and the rest developed ovotestes with varying degrees of testicular tissue, often minimal. WMISH revealed a range of *Sox9* expression profiles, consistent with XY ovary and ovotestis formation (Figures 4H and 4I). There were large numbers of *Stra8*-positive cells in heterozygous XY gonads (Figures 4M and 4N), again indicative of activation of the ovarian pathway due to disruption to the core testis-determining gene regulatory network. Thus, as previously reported for *Map3k4*, *Gadd45γ* is a dosage-sensitive testis-determining gene.

We probed the regulatory relationship between GADD45γ and MAP3K4 in testis determination further using a gain-of-function genetic approach. First, we generated a mouse line transgenic for a functional *Map3k4* bacterial artificial chromosome (BAC) clone (see Experimental Procedures). This BAC transgenic line was capable of rescuing the XY gonadal sex reversal caused by complete loss of endogenous *Map3k4* (data not shown). Assessment of the levels of *Map3k4* transcript in BAC transgenic gonads at 11.5 dpc using qRT-PCR revealed an approximately 3.5-fold increase over wild-type (Figure 4P). We then performed crosses demonstrating that transgenic overexpression of *Map3k4* rescues abnormalities in testis determination caused by haploinsufficiency of *Gadd45γ* on B6-Y^AKR^ (compare Figures 4H and 4I with Figure 4J and Figures 4M and 4N with Figure 4O). These data show that deficiencies in GADD45γ function can be compensated by increased expression of MAP3K4, supporting a model in which the two molecules interact in the same cellular pathway during testis determination.

### A Tissue-Specific Differentially Methylated Region of *Sry* in Embryonic Gonadal Somatic Cells Is Unaffected by Loss of *Gadd45γ*

While transcription factors and cofactors have been associated with control of mouse *Sry* expression (reviewed in Warr and Greenfield, 2012), there have been few reports concerning regulatory elements of the *Sry* locus itself required for its functional gonadal expression (Albrecht and Eicher, 2001). However, any such elements must be present in the genomic fragment that has been demonstrated to cause XX gonadal sex reversal in transgenic mice (Koopman et al., 1991). An *Sry* “promoter region,” comprising approximately 8 kb 5′ of the translational start site of *Sry*, is sufficient to drive green fluorescent protein (GFP) reporter gene expression with a timing and cellular specificity similar to that of the endogenous gene (Albrecht and Eicher, 2001). More recently, an approximately 600 bp region immediately 5′ to the transcription start site of the linear gonadal *Sry* transcript has been reported to contain a number of CpG dinucleotides that exhibit differential methylation of the cytosine in different embryonic tissues, a so-called tissue-dependent differentially methylated region (T-DMR) (Nishino et al., 2004) (Figure S2A). This region is hypomethylated in somatic cells of the gonad, in contrast to the embryonic liver in which it is hypermethylated.

A number of strands of evidence indicate that GADD45 proteins also act during active DNA methylation at specific loci, possibly due to their role in base excision repair during the demethylation process (reviewed in Niehrs and Schäfer, 2012). Because of this functional role, we examined the *Sry* T-DMR in somatic cells derived from wild-type, *Gadd45γ*^−*/*−^ and *Map3k4*^−*/*−^ embryonic gonads at 15–16 ts (Nishino et al., 2004). We first confirmed, using qRT-PCR, that the cell fraction enriched for somatic cells had greatly reduced expression of *Oct4* in contrast to whole-gonad samples (data not shown). We then validated the existence of the T-DMR by bisulphite sequencing of the *Sry* promoter region, encompassing six CpG dinucleotides, in the purified gonadal somatic cells and embryonic limb cells at 11.5 dpc. This analysis revealed significant hypomethylation in gonadal somatic cells in contrast to hypermethylation of this region in limb cells, which do not express *Sry* (Figure S2B). Thus, hypomethylation of this region is associated with *Sry* expression in vivo. However, bisulphite analysis of this region in somatic cells derived from *Gadd45γ*^−*/*−^ and *Map3k4*^−*/*−^ embryonic gonads revealed no significant increase in methylation of the *Sry* promoter when compared to wild-type gonads (Figure S2C). From this observation, we conclude that GADD45γ is not required for establishment of a hypomethylated region in the vicinity of the *Sry* promoter region.

### Disruption to p38 MAPK Signaling in *Gadd45γ*^−*/*−^ and *Map3k4*^−*/*−^ Embryonic Gonads at 11.25 dpc

**Figure 5:**
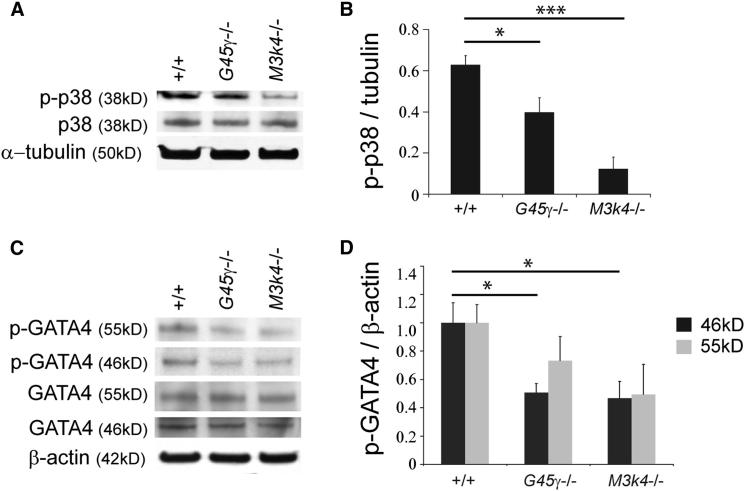
Reduced Phosphorylation of p38 MAPK and GATA4 in Embryonic Gonads Lacking GADD45γ or MAP3K4 (A–C) Immunoblotting of subdissected XY gonad samples at the 15–16 ts stage (A and C). Relative quantitation of phospho-p38 normalized to the α-tubulin loading control (B). (D) Relative quantitation of phospho-GATA4 normalized to the β-actin loading control. Error bars, SEM.

We have previously examined the tissue distribution of a variety of MAP2Ks and MAPKs, along with their activated (phosphorylated) forms, in gonads from wild-type and *Map3k4*^−*/*−^ embryos at 11.5 dpc by immunostaining (Bogani et al., 2009). These studies did not establish any obvious misregulation of MAPK signaling in the gonads of *Map3k4*-deficient embryos. However, no quantitative analyses were performed in these earlier studies, and gonads were not studied prior to 11.5 dpc. Here, we modified the analysis of MAPK signaling to encompass an earlier and, most likely, more relevant time point for analyzing the cause of disrupted *Sry* expression. In addition, we performed protein quantitation by western blotting of lysates from gonads dissected away from the attached mesonephros. These analyses revealed significant reduction in the levels of phospho-p38 MAPK (p-p38) in *Map3k4*^−*/*−^ gonads at around 11.0–11.25 dpc (15–16 ts) (Figures 5A and 5B). Similarly, p-p38 levels in *Gadd45γ* ^−*/*−^ gonads were also significantly reduced at the same stage, although to a lesser extent (Figures 5A and 5B). This difference may reflect the consequences of constitutive loss of MAP3K4 in *Map3k4*^−*/*−^ gonads and the more restricted disruption to MAP3K4 function in somatic cells of *Gadd45γ*^−*/*−^ gonads. Overall, these data indicate a failure to activate p38 MAPK to wild-type levels in both *Gadd45γ*^−/−^ and *Map3k4*^−*/*−^ gonads, implicating p38 signaling in the regulation of *Sry* expression and testis determination.

### Loss of Both p38α and p38β Causes Embryonic XY Gonadal Sex Reversal

**Figure 6:**
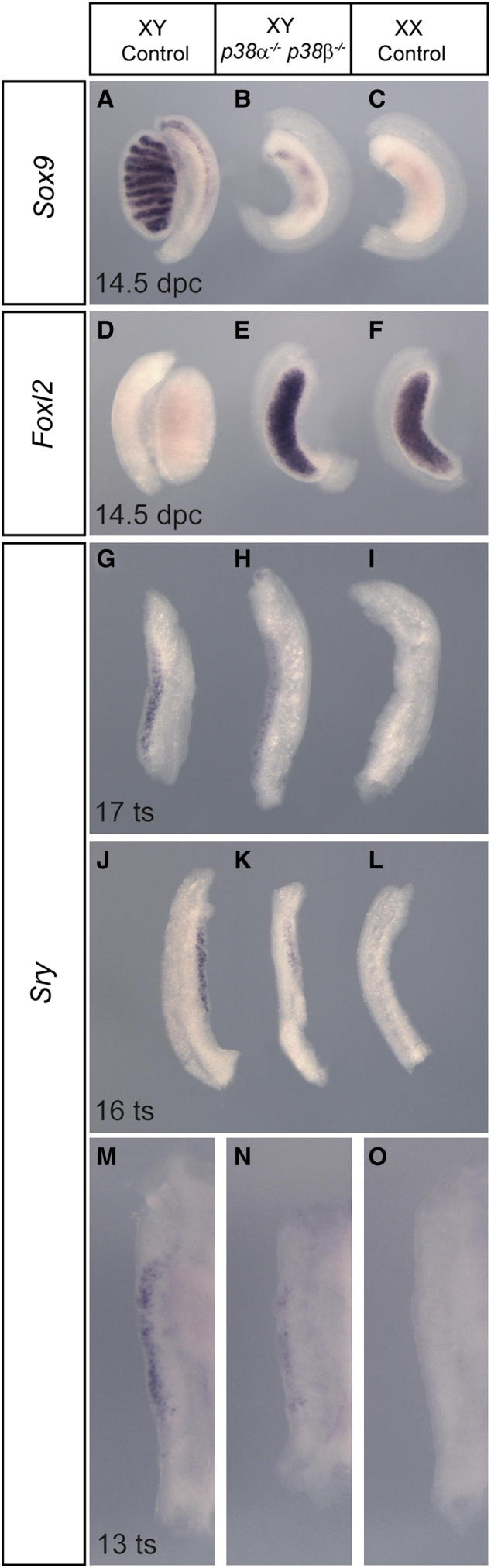
XY Sex Reversal in Embryonic Gonads Lacking Both p38α and p38β MAPKs (A–F) WMISH of XY double-knockout embryos reveals loss of *Sox9* expression (A–C) and gain of *Foxl2* expression (D–F) in gonads at 14.5 dpc, when compared with control genotypes. (G–O) *Sry* expression is reduced in doubly mutant XY embryonic gonads at 13 ts (N), 16 ts (K), and 17 ts (H), in comparison to XY controls (M, J, and G, respectively). Control embryos lacked at most one copy of *p38a*. Such control XY gonads never exhibited sex reversal at 14.5 dpc. However, loss of *p38α* or *p38β* alleles in the control gonads used here may possibly reduce the observable differences in *Sry* levels between control and doubly homozygous gonads.

The reduction in p38 MAPK activation in GADD45*γ*- and MAP3K4-deficient embryonic gonads described earlier leads to the hypothesis that appropriate activation of p38 MAPK signaling is a requirement for testis determination in mice. The p38 MAPK family is encoded by four distinct genes: *p38α* (*Mapk14*), *p38β* (*Mapk11*), *p38γ* (*Mapk12*), and *p38δ* (*Mapk13*). All four isoforms are expressed in the developing XY and XX gonads between 11.5 and 13.5 dpc (Ewen et al., 2010). We, and others, have previously shown that embryonic gonads are sensitive to the commonly used chemical inhibitors of p38 MAPK, SB203580, and SB202190, which cause somatic and germ cell sex reversal in ex vivo organ culture experiments (Bogani et al., 2009; Ewen et al., 2010). In order to avoid potential off-target effects arising from the use of inhibitors but also overcome possible functional redundancy, we tested the requirement for p38 MAPKs in testis determination genetically. Mice lacking both p38γ and p38δ are reported to be viable and fertile (Sabio et al., 2005). While this does not entirely rule out a role for these two genes in gonad development, we chose to focus on examining gonadogenesis in XY embryos lacking both p38α and p38β on a predominantly B6 background. This focus is also warranted because p38α and p38β are around 70% identical at the amino acid level, have similar substrate specificities, and are both sensitive to SB203580 and SB202190 (Cuadrado and Nebreda, 2010). Mice lacking p38β are reported to be viable and fertile (Beardmore et al., 2005), and this was confirmed in our studies. Mice lacking p38α exhibit early embryonic lethality due to placentation defects (Adams et al., 2000), which would preclude an analysis of gonad development. Therefore, we exploited a strategy in which *p38α* ablation is restricted to epiblast-derived tissues by the use of *Sox2*-*Cre*-mediated deletion of floxed *p38α* alleles, as previously described (del Barco Barrantes et al., 2011). A breeding strategy was then used to combine these alleles (see Experimental Procedures). Embryos homozygous for both the *p38α* and *p38β* null alleles exhibit cardiovascular abnormalities and spina bifida (del Barco Barrantes et al., 2011). Remarkably, examination of gonads from these embryos also revealed complete XY sex reversal at 14.5 dpc (Figure 6). The XY gonads had an overt ovarian morphology, and WMISH confirmed loss of *Sox9* (Figures 6A–6C) and gain of *Foxl2* expression (Figures 6D–6F). Notably, an XY embryo lacking p38α alone did not exhibit overt defects in testis determination, while embryos lacking both copies of *p38α* and only one of *p38β* did exhibit gonadal sex reversal (data not shown). Significantly, examination of *Sry* expression at 11.0 dpc (13–14 ts) and 11.5 dpc (16–17 ts) in double-knockout embryos revealed a reduction in gonadal *Sry* expression when compared to controls (Figures 6G–6O). We conclude that simultaneous loss of both p38α and p38β causes disruption to *Sry* expression and XY embryonic gonadal sex reversal.

### Reduced Levels of Phospho-GATA4 in *Map3k4*^−*/*−^ and *Gadd45γ*^−*/*−^ Embryonic XY Gonads

Several transcriptional regulators are implicated in regulation of *Sry* based on the reduction of *Sry* expression in their absence (reviewed in Sekido and Lovell-Badge, 2009 and Warr and Greenfield, 2012). We hypothesized that MAPK signaling may be required to activate one or more of these transcriptional regulators by phosphorylation. Immunostaining with antibodies detecting a number of such putative regulators of *Sry* expression revealed no overt differences between wild-type and *Gadd45γ-*deficient gonads (data not shown). In most cases, antibodies that specifically detect phosphorylated forms of such proteins and can be reliably used for quantitation by western blot are not available. However, such an antibody was available in the case of the known testis-determining protein, GATA4, which is implicated in the regulation of *Sry* expression (Tevosian et al., 2002). Phosphorylation of GATA4 can enhance its DNA-binding potency and increase expression of GATA4-regulated transcripts. In addition, GATA4 can be directly phosphorylated at serine 105 (S105) by ERK1/2 and p38 MAPKs (Charron et al., 2001; Liang et al., 2001; Tenhunen et al., 2004). Because tissue immunostaining is not appropriately quantitative, we again analyzed the expression of GATA4 and phospho-GATA4 (p-GATA4: S105) by western blot analysis of protein extracts from embryonic XY gonads at 15–16 ts. While there was some variability in the levels of p-GATA4 detected across samples, we saw a consistent association between loss of *Gadd45γ* or *Map3k4* and a reduction of p-GATA4. In contrast, no significant differences existed in levels of GATA4 (Figures 5C and 5D). Thus, GATA4 may be a direct target of the GADD45γ/MAP3K4/p38 MAPK signaling pathway in the embryonic gonad, and diminution of GATA4 phosphorylation may contribute to disruption to *Sry* expression observed in mutants described here.

## Discussion

**Figure 7:**
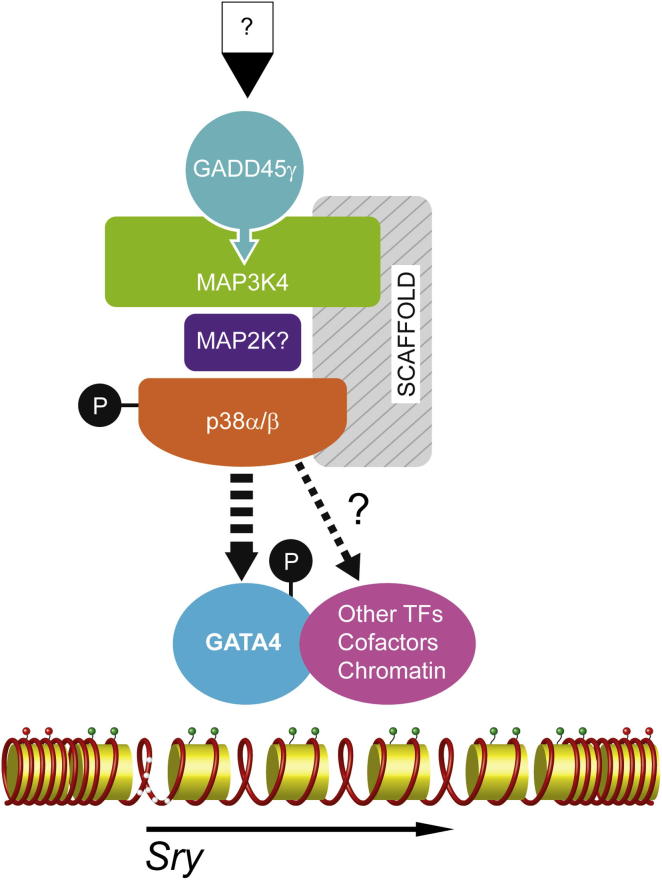
Summary of Proposed Interactions between GADD45γ, MAP3K4, p38 MAPK, GATA4, and *Sry* in Testis Determination The signals regulating the expression of *Gadd45γ* shortly after gonad formation (indicated by a question mark at the top) are unknown. Based on data reported here and described in the supporting literature, we propose that GADD45γ activates MAP3K4 in supporting cell precursors of the developing gonad from around 10.5 dpc. MAP3K4 activates p38α and p38β (indicated by a circled P) through an as yet uncharacterized MAP2K. This MAPK phosphorelay module, perhaps in the context of an unidentified scaffold protein, results in the direct or indirect activation of GATA4 by phosphorylation (dashed arrow) and subsequent expression of *Sry* in XY supporting cells. In addition to DNA demethylation of its promoter region (white circles on DNA strand), which does not require GADD45γ/MAP3K4, timely *Sry* expression may require chromatin marks also established by this signal transduction pathway (green circles). Additional targets of this MAPK cascade in the developing gonad are not excluded but remain to be identified, possibly comprising other chromatin-associated transcription factors (TFs), transcriptional cofactors, and chromatin-modifying enzymes that alter chromatin configuration at *Sry* (and/or other loci).

We show here that *Gadd45γ*, which encodes a protein first associated with the cellular stress response, and *p38α/p38β*, which encode paradigmatic stress-activated signaling molecules, are primary autosomal testis-determining genes. We also provide evidence supporting the existence of a molecular pathway linking GADD45γ, MAP3K4, and p38 MAPKs that functions in vivo during a key event in mammalian organogenesis: cell fate determination in the developing gonad (summarized in Figure 7). XY embryos lacking GADD45γ or both p38α and p38β phenocopy those lacking MAP3K4 in exhibiting gonadal sex reversal caused by a reduction in the levels of *Sry* expression at a critical stage of testis determination. Moreover, sex reversal in all three mutants studied is associated with reduction in the levels of p38 MAPK signaling. Embryos lacking a single copy of both *Gadd45γ* and *Map3k4* also exhibit a small delay in testis determination as indicated by activation of the ovarian pathway at the gonadal poles. This indicates genetic interaction between the two loci and the existence of a genetic pathway that is disrupted by this dual loss. Rescue of XY gonadal sex reversal associated with *Gadd45γ* haploinsufficiency on C57BL/6-Y^AKR^ by a *Map3k4* BAC transgene also supports the model of a dosage-dependent interaction between the two loci during testis determination. These data, along with studies of GADD45γ in other contexts, suggest that the simplest explanation of the sex-reversal phenotypes described here involves regulation of MAPK signaling by GADD45γ in the early gonad to control the timing of *Sry* expression. Failure to activate MAP3K4/p38 MAPK signaling results in a significant shift in the temporal dynamics of *Sry* expression, characterized by delayed *Sry* expression, failure of Sertoli cell fate determination, and consequent activation of ovary-determining genes. While the observed early disruption to *Sry* expression in mutants of this pathway is sufficient to explain downstream loss of other testis lineage markers, including *Sox9*, we cannot exclude later functions for proteins in this, or closely related, signal transduction pathways in testis determination and differentiation, not least due to the role of FGF signaling in this process (Kim et al., 2006). These issues are being addressed by conditional gene targeting.

One significant contribution of the present study relates to the restricted expression profile of *Gadd45γ* that, in contrast to *Map3k4*, locates the primary site of action of this pathway in somatic cells in the interior of the newly formed gonad. GADD45γ appears to offer specificity to the employment of MAPK signaling in the developing gonad, activating it in a spatiotemporal fashion required for somatic cell differentiation. Notwithstanding the absence of any significant sexual dimorphism, the dynamic spatiotemporal profile of *Gadd45γ* expression is remarkably similar to that of *Sry,* both in terms of the center-to-pole spatial expansion and the loss of expression around 12.5 dpc. However, the molecular basis of this downregulation remains unclear. Our data suggest a direct role for GADD45γ in the regulation of *Sry* expression in pre-Sertoli cells via activation of p38 MAPK. We also describe data indicating that GATA4, a protein that is known to be activated by MAPK signaling and has itself been implicated in the regulation of *Sry* expression, is not appropriately phosphorylated in *Gadd45γ* - and *Map3k4*-deficient gonads, offering a mechanistic link between defective p38 MAPK signaling and misregulation of *Sry* expression (Gierl et al., 2012 [this issue of *Developmental Cell*]). Further work, including the phenotypic analysis of mice with GATA4 variants incapable of phosphorylation, will be required to substantiate a causal link between p38 MAPK signaling, GATA4 activation and the timing of *Sry* expression in testis determination. Undoubtedly, other targets of MAP3K4/p38 MAPK signaling, also potentially involved in regulation of *Sry*, remain to be identified in the embryonic gonad in vivo (Figure 7), although systematic identification of these remains technically daunting, given limiting tissue sources and tissue complexity.

It will be important to determine the signals regulating expression of *Gadd45γ* itself in XY and XX gonads and whether its expression is dependent on other, well-characterized gonadal transcription factors such as WT1, SF1, or insulin-like growth factor receptors. The expression of *Gadd45γ* and other elements of the MAPK pathway in XX gonads is likely to contribute to the capacity of such gonads to support expression of *Sry* transgenes in different contexts (Albrecht and Eicher, 2001; Koopman et al., 1991). It will also be important to determine whether mutations in *GADD45γ*, *p38α* (*MAPK14*) and *p38β* (*MAPK11*) can disrupt human testis determination, as is the case for the gene encoding the MAPK kinase kinase, *MAP3K1* (Pearlman et al., 2010). To our knowledge, no role exists for GADD45 proteins in activating MAP3K1. It is also worth noting that the p38 MAPK-deficient embryos examined here do not complete development (del Barco Barrantes et al., 2011), suggesting that some mutations in human testis-determining *MAPK* genes may result in nonviability.

Previous studies demonstrated that all three GADD45-like proteins (α, β, and γ) activate MAP3K4 (Takekawa and Saito, 1998). Subsequent studies showed that GADD45-mediated activation of MAP3K4 requires binding of GADD45 to the N terminus of MAP3K4, which relieves autoinhibition by promoting MAP3K4 dimerization and autophosphorylation (Miyake et al., 2007). Notably, MAP3K4 mediates the action of GADD45β and GADD45γ in activating p38 MAPK in T cells and stimulating production of the cytokine, IFNγ (Chi et al., 2004). This last report is significant in the context of this study in supporting a role for a GADD45β/GADD45γ/MAP3K4 pathway in T cells, although the study did not establish T cell defects in mice heterozygous for *Map3k4* and *Gadd45*γ null mutations. Our data describe a requirement for GADD45γ/MAP3K4/p38 MAPK in vivo during embryonic cell fate specification.

Several studies suggest a role for GADD45 proteins in active DNA demethylation, probably via their recruitment of the DNA repair machinery to specific target sites to permit excision of methylated nucleotides and replacement with unmethylated residues (Niehrs and Schäfer, 2012). However, we found no evidence of increased methylation at a series of CpG nucleotides in the vicinity of the *Sry* promoter in the absence of GADD45γ or MAP3K4. We cannot exclude the possibility that GADD45γ is required for demethylation at other regions of *Sry* or other loci that function in the regulation of *Sry* expression. Alternatively, GADD45γ may function to activate MAP3K4 in pathways unrelated to DNA methylation, perhaps involving other epigenetic modifications, as reported in stem cell contexts (Abell et al., 2011). It is also worth noting that the established molecular roles of GADD45 proteins in active DNA demethylation and the regulation of MAPK signaling may not be independent functions. It has been proposed that the physical interaction between GADD45, AID/Apobec, and MBD4 associated with active DNA demethylation may reflect the role that GADD45 plays in activating these associated enzymes by positively regulating downstream MAPKs (Rai et al., 2008).

Data exist linking p38 MAPKs with regulation of gene expression via recruitment to chromatin. For example, p38 MAPK is actively recruited to chromatin via its interaction with transcription factors (Ferreiro et al., 2010) and can facilitate chromatin remodelling mediated by the SCRAP complex via H2A.Z exchange (Cuadrado et al., 2010). Such reports of p38 MAPK-mediated changes to chromatin are significant in the context of a putative direct role in the regulation of *Sry* expression. The mammalian Y chromosome is the most heterochromatic chromosome and is intensely stained by the repressive chromatin mark, histone H3 trimethylated at lysine 9 (H3me3K9) (Bulynko et al., 2006). The removal of such marks and their replacement by positive variants may be a prerequisite of timely *Sry* expression in pre-Sertoli cells. Future studies of the epigenomics of testis determination should shed light on these questions.

## Experimental Procedures

### Mouse Strains and Genotyping

All animal experimentation was approved by the local ethical review process and performed with licensed approval from the UK Home Office (PPL 30/2877). *Gadd45γ* knockout mice have been previously described (Hoffmeyer et al., 2001). In these mice, most of the coding sequence of *Gadd45γ* has been replaced by a selection cassette. *Gadd45γ* heterozygotes were maintained on C57BL/6J and genotyped by quantitative PCR (primers available upon request). *Map3k4* knockout mice were maintained on C57BL/6J and genotyped as described (Bogani et al., 2009).

Mice carrying *p38α(flox)*, *p38β* null, and *Sox2Cre* alleles have been previously described (del Barco Barrantes et al., 2011). Doubly homozygous embryos were generated by crossing *p38α*^*Δ/+*^, *p38β*^*+/*−^, *Sox2Cre* males with *p38α*^*flox/flox*^, *p38β*^*+/*−^ females or *p38α*^*Δ/+*^, *p38β*^−*/*−^, *Sox2Cre* males with *p38α*^*flox/flox*^, *p38β*^−*/*−^ females.

*W*/+ mice were intercrossed to generate *W/W* embryos. The *W* allele of *c-kit* contains an intronic point mutation causing exon skipping and a 234 base pair (bp) deletion of the mRNA (Hayashi et al., 1991). The mutation was detected by sequencing of genomic PCR products. Embryos were chromosomally sexed by a PCR assay (Warr et al., 2009).

### Generation of Embryos and Expression Analyses

Noon on the day of the copulatory plug was counted as 0.5 dpc. Embryos were staged accurately by the number of ts. WMISH of embryonic tissues was performed as previously described (Bogani et al., 2009). Probes for *Foxl2* and *Gata4* were generated by RT-PCR.

### Immunohistochemistry and Western Blotting

The following antibodies were used: SRY, SOX9, FOXL2 (all kind gifts from Dagmar Wilhelm and Peter Koopman), SOX9 (Millipore), AMH (Santa Cruz), GATA4 (Santa Cruz), phospho-GATA4 (pS105, Abcam), p38 (Cell Signaling), phospho-p38 (Cell Signaling), α-tubulin (Hybridoma Bank, University of Iowa), β-actin (Abcam), and Ki-67 (Vector Lab). After immunostaining, sections were counterstained with DAPI and cells were visualized using a Zeiss Axiophot2.

For western blot analysis, gonads were dissected in phosphate-buffered saline (PBS) containing phosphatase and protease inhibitors (Roche). Pools of six gonads were homogenized in Laemmli buffer with 0.025 μM EDTA, vanadate (1×), and β-mercaptoethanol. Samples were sonicated for 15 cycles of 15 s with a water-bath sonicator and analyzed by SDS-PAGE. Quantitation was performed by analysis of four independent blots.

For analysis of cell proliferation, total cell counts (GATA4- and Ki-67-positive) were performed on three gonads of each genotype (four nonadjacent, longitudinal sections from each gonad).

### Bisulphite Sequencing

Embryonic gonads were dissociated in Dulbecco's modified Eagle's medium: Nutrient Mixture F-12 containing collagenase A (5 mg/ml) at 37°C for 30 min. DNA and RNA were extracted after removal of germ cells using the differential cell attachment methodology of Nishino et al., 2004. This allowed quantitative RT-PCR analysis of transcription and bisulphite conversion of genomic DNA (gDNA) using a micro-DNA/RNA kit (QIAGEN). Bisulphite conversion was performed on 100 ng of gDNA using EpiTect Bisulfite kit (QIAGEN). PCR reactions were performed on 1 μl of converted gDNA using 2× Thermo-Start Reddy mix (Abgene) with 2 μM each of primers. A first round of PCR was performed with *Sry* primers 5′-AAGGAAATGATAGTAGTTTTGATT-3′ and 5′-ACCAAAATATACTTATAACAAAAATTTTAA-3′; a second round of PCR was then performed on 1 μl of first-round product using the primers 5′-AAGGAAATGATAGTAGTTTTGATT-3′ and 5′-TCAAACAAAACCCCTCAAATTTATAAA-3′.

### Generation of *Map3k4* BAC Transgenic Lines

A 178.7-kb NOD/MrkTac BAC clone (bQ279c06 (c06)) containing the complete *Map3k4* transcription unit and flanking DNA regions, but no other complete gene loci, was sourced from the Centre for Applied Genomics, Toronto, Ontario, Canada. BAC DNA was prepared and injected into C57BL/6J 1-cell embryos to produce transgenic founders (Gardiner and Teboul, 2009).
