# Supplementary material for: Gadd45γ and Map3k4 Interactions Regulate Mouse Testis Determination via p38 MAPK-Mediated Control of Sry Expression
Source: Dev Cell. 2012 Nov 13;23(5):1020–31. doi: 10.1016/j.devcel.2012.09.016 (PMC3526779; doi:10.1016/j.devcel.2012.09.016)
Supplement: Document S1. Figures S1 and S2 [file mmc1.pdf]

Developmental Cell, Volume *23*

Supplemental Information

Gadd45γ and *Map3k4* Interactions

Regulate Mouse Testis Determination via p38

MAPK-Mediated Control of *Sry* Expression

Nick Warr, Gwenn-Aël Carre, Pam Siggers, Jessica Vitos Faleato, Rachel Brixey, Madeleine Pope, Debora Bogani, Melissa Childers, Sara Wells, Cheryl L. Scudamore, Marianna Tedesco, Ivan del Barco Barrantes, Angel R. Nebreda, Paul A. Trainor, and Andy Greenfield

INVENTORY OF SUPPLEMENTARY FIGURES

Figure S1, related to Fig. 3. Somatic cell proliferation is not perturbed in XY *Gadd45γ*–deficient embryonic gonads at approximately 11.0 dpc.

Figure S2, related to Fig. 3. Analysis of the methylation status of six CpGs at the *Sry* promoter.

Both of these supplementary Figures are related to the main Fig. 3, being analyses of potential defects in *Gadd45γ* $^{-/-}$ and *Map3k4* $^{-/-}$ gonads. However, in contrast to the main Figures, data described in these supplementary Figures do not identify defects i.e. no differences are detected between mutant and control gonad samples.

SUPPLEMENTARY FIGURE LEGENDS

Figure S1. Somatic cell proliferation is not perturbed in XY *Gadd45γ*–deficient embryonic gonads at approximately 11.0 dpc. Immunohistochemistry with GATA4 (gonadal somatic cells) and Ki67 (cell proliferation marker) allows assessment of somatic cell proliferation in wild-type (A) and *Gadd45γ*–deficient gonads (B). Cell counts reveal no significant difference in the proportion of GATA4-positive cells that are also Ki67-positive between wild-type (+/+) and mutant (-/-) gonads (C).

Figure S2. Analysis of the methylation status of six CpGs at the *Sry* promoter. A 526bp region containing six published CpGs was amplified (A). Relative positions to the transcription start site of *Sry* are indicated. (B-C) Representative bisulphite sequencing reads indicating methylation status of CpGs. Wildtype extra-gonadal (limb) and gonadal samples (at different stages of development) (B). Comparison of wild-type and mutant gonadal samples at 15/16ts stage (C). Open and filled circles indicate unmethylated and methylated, respectively.

Fig.S1

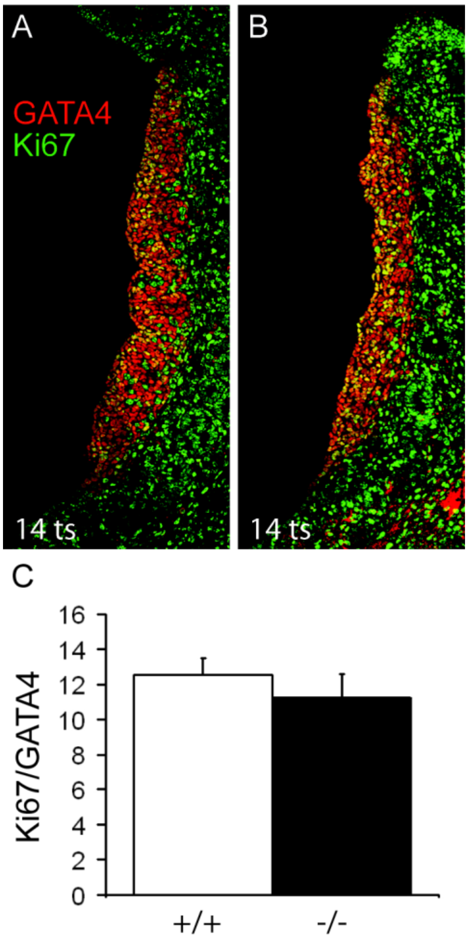

Fig.S2

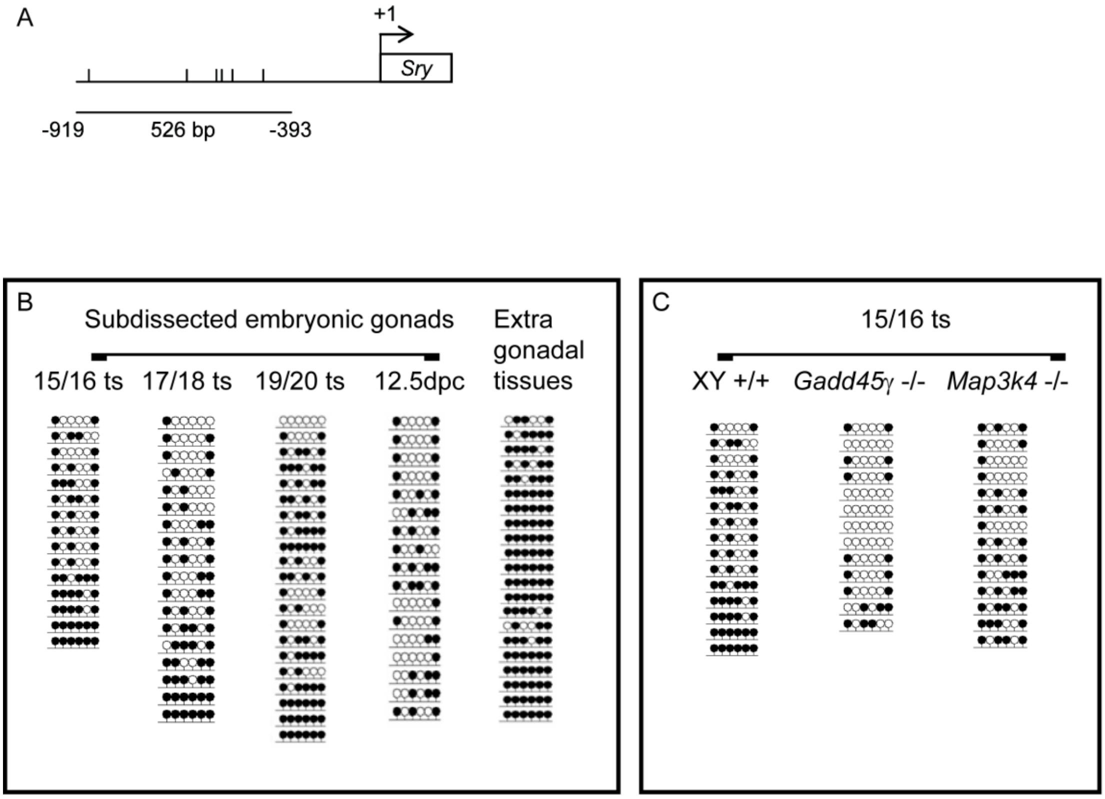
A
+1
Sry
-919
526 bp
-393
B
Subdissected embryonic gonads
15/16 ts
17/18 ts
19/20 ts
12.5dpc
Extra gonadal tissues
C
15/16 ts
XY +/+
Gadd45γ -/-
Map3k4 -/-
